# Stocks of paracetamol products stored in urban New Zealand households: A cross-sectional study

**DOI:** 10.1371/journal.pone.0233806

**Published:** 2020-06-01

**Authors:** Eeva-Katri Kumpula, Pauline Norris, Adam C. Pomerleau

**Affiliations:** 1 National Poisons Centre, Department of Preventive and Social Medicine, University of Otago, Dunedin, New Zealand; 2 Division of Health Sciences, Centre for Pacific Health Va’a o Tautai, University of Otago, Dunedin, New Zealand; Medical University Graz, AUSTRIA

## Abstract

**Background:**

Intentional self-harm is a common cause of hospital presentations in New Zealand and across the world, and self-poisoning is the most common method of self-harm. Paracetamol (acetaminophen) is frequently used in impulsive intentional overdoses, where ease of access may determine the choice of substance.

**Objective:**

This cross-sectional study aimed to determine how much paracetamol is present and therefore accessible in urban New Zealand households, and sources from where it has been obtained. This information is not currently available through any other means, but could inform New Zealand drug policy on access to paracetamol.

**Methods:**

Random cluster-sampling of households was performed in major urban areas of two cities in New Zealand, and the paracetamol-containing products, quantities, and sources were recorded. Population estimates of proportions of various types of paracetamol products were calculated.

**Results:**

A total of 174 of the 201 study households (86.6%) had at least one paracetamol product. Study households had mostly prescription products (78.2% of total mass), and a median of 24.0 g paracetamol present per household (inter-quartile range 6.0–54.0 g). Prescribed paracetamol was the main source of large stock. Based on the study findings, 53% of New Zealand households had 30 g or more paracetamol present, and 36% had 30 g or more of prescribed paracetamol, specifically.

**Conclusions:**

This study highlights the importance of assessing whether and how much paracetamol is truly needed when prescribing and dispensing it. Convenience of appropriate access to therapeutic paracetamol needs to be balanced with preventing unnecessary accumulation of paracetamol stocks in households and inappropriate access to it. Prescribers and pharmacists need to be aware of the risks of such accumulation and assess the therapeutic needs of their patients. Public initiatives should be rolled out at regular intervals to encourage people to return unused or expired medicines to pharmacies for safe disposal.

## Introduction

Intentional self-poisoning (ISP) where too much of a medication or other substance is taken by a person for the purpose of self-harm is a common type of injury treated in Emergency Departments in New Zealand, with paracetamol (acetaminophen) commonly involved [1]. Paracetamol is commonly encountered in exposures also in, for example, Australia [2–4], Ireland [5–7], England [8], Canada [9, 10], and the United States [11]. Poisoning fatalities in general occur at an annual rate of 7.1/100,000 population in New Zealand, and 71% of them are intentional [12]. Hospital admissions due to poisoning occur at an annual rate of 115.4/100,000 in New Zealand, and again 65% of such presentations are due to ISP [12], with an annual rate of 70.86 hospital presentations per 100,000 population for suspected ISP [1]. Rates of hospital presentations may not be directly comparable internationally due to differences in definitions and methodologies, e.g. what length of stay constitutes an ‘admission’ which is captured in a given dataset, and which case definition is used for ISP or suicide. World Health Organization international data on suicide deaths show that New Zealand has roughly comparable rates to e.g. Australia, France, the United States, Czech Republic, and Ireland [13]. Young people, those living in deprived neighborhoods, and indigenous Maori have the highest rates of suicide [13].

The decision to engage in ISP and the choices of substances are often very impulsive, depending on what is available to the person at that particular moment [14]. The person’s own prescription medicines are a common source of substances for ISP [15]. Paracetamol may be chosen due to ease of access, and because people have a limited understanding of its effects in overdose [16, 17]. In adults, acute paracetamol overdoses of 10 g or more may be associated with acute liver injury and are therefore of concern, and overdoses above 30 g may necessitate enhanced management such as the use of higher doses of the antidote [18]. As impulsivity plays an important part in choices of agents taken in ISP, any stock immediately available in the home in excess of 10 g could be of concern, and unnecessary accumulation of large stock should be avoided. Efforts to reduce these need to be informed by evidence about current household stocks and how they were obtained. Availability of stocks of medicines in the home can facilitate ISP. However, literature on household storage of medicines is limited, and much of it is carried out in developing countries [19–21]. In New Zealand, the Medications in Everyday Life project explored places of storage but did not quantify amounts stored [22].

In New Zealand, paracetamol can be obtained from pharmacies by prescription (up to 360 g in one dispensing), and without a prescription in packages of up to 50 g of the immediate-release and almost 64 g of the modified-release product in a single purchase. Products containing up to 10 g paracetamol per package can also be purchased from supermarkets and other non-pharmacy retailers, but there is no limit to how many packs can be purchased at a time.

This cross-sectional study aimed to determine how much paracetamol was present in New Zealand households, and from where it was obtained. The purpose of the study was not to determine what the indications for paracetamol use might have been, or whether stocks were appropriate for a particular situation, but rather simply how much was present and therefore easily available for impulsive overdoses. These aims were achieved and the findings have implications for policy and practice to reduce ISP.

## Materials and methods

A survey study with random, clustered sampling of consenting household members in two cities in New Zealand was designed. Ethical approval was obtained from the University of Otago Human Research Ethics Committee (ref: D19/171). Written, informed consent was obtained from participants. As people aged 16 and over are able to legally give informed consent to participate in research and decide on their own medical care in New Zealand [23, 24], anyone meeting this age criterion was eligible to participate.

### Sampling

Data were collected from June to October 2019. A total of 201 households in 40 meshblocks in two Major Urban Areas (MUAs; areas of 100,000 or more residents) of Dunedin and Auckland were sampled. Meshblocks are Statistics NZ’s smallest geographic unit, and roughly correspond to a city block or part of it [25]. The two cities were chosen to obtain a convenience sample due to practical reasons, including a large city with many ethnicities represented (Auckland; 1.5 million people), and a smaller regional city (Dunedin; 130,000 people). Random cluster-sampling of 20 meshblocks in each city was performed by deprivation level, where all eligible MUA meshblocks were stratified by their New Zealand Deprivation Index 2013 (NZDep2013) index scores [26], which describe the level of area deprivation by taking into account multiple relevant area and household variables [27]. Six meshblocks were randomly selected from each city from NZDep2013 8–10 meshblocks (most deprived), eight from NZDep 4–7, and 6 from NZDep2013 1–2 (least deprived), for a total of 40 meshblocks. This was done to obtain a sample that would be representative of the general New Zealand population by levels of deprivation. This does not guarantee full representativeness, however, as the sample was urban only, and because other factors besides area deprivation may affect representativeness. There were 11,193 meshblocks in total eligible to be randomly chosen, and 20/10,798 eligible (0.2% of all eligible) were chosen from Auckland, and 20/1,115 eligible (1.8% of all eligible) were chosen from Dunedin. Each meshblock was sampled by starting from a random end of the street and then tossing a dice to choose a house to approach, and repeating this until either five households were recruited or there were no more households to sample.

### Recruitment and data collection

Data were collected by two research assistants (RAs) working together as a pair in each city (a total of four people). The RAs were university graduates trained by the first author to safely approach households and present the study to the householder. They were also trained to facilitate data collection by showing images of paracetamol products available in New Zealand, especially combination products which people may not realize contain paracetamol [28], and to encourage the participant to bring their own medicines and those shared by the household, to be recorded. The RAs were also specifically trained to read New Zealand prescription labels and packaging markings to obtain the information needed for the study.

Data were collected on all days of the week during daylight hours (9am to 6pm) to maximize people’s chances of being home when their meshblock was sampled, though only on one day in total per meshblock. RAs knocked on the doors of domiciles in each meshblock to be sampled, chosen by tossing a dice as described. Inclusion criteria: person present and usually residing in a domicile in a meshblock which was sampled, and aged 16 or over. Exclusion criteria: not able to give informed consent (intoxicated, aggressive, otherwise not safe to approach–nobody was excluded for this reason).

Household members aged 16 years and over were eligible to participate, and if consent was obtained, basic demographics were collected about the household (number of people usually residing in the household, their age, sex, ethnicity). Participants were then shown images of paracetamol-containing products (sole and combination), and requested to bring out all paracetamol products of their own, and any that were shared by the household in communal areas of the domicile. Private stock of any other residents of the household who were not present and were therefore unable to consent was not recorded for ethical reasons. The RAs never entered any domicile to look for medicines but stayed outside–it was fully up to the participant to bring all relevant medicines out. Suppositories were excluded from the study as they are rarely used in New Zealand, and not generally used for self-harm. If there were paracetamol products present, product type, strength, expiry date, purchase date and means of obtaining (by prescription, pharmacy over-the-counter [OTC], other retailer [i.e. not a pharmacy; e.g. supermarket, petrol station], other, unknown) were recorded. Participants received a NZ$10 voucher (~US$6) as a token of appreciation for their time spent on the study. No details were collected from the households that declined to take part.

### Analysis

A household was chosen as the unit of analysis, regardless of how many people resided in it. Amounts of paracetamol stock were calculated by summing up all milligrams per dosing unit present, to reflect paracetamol weight only, in grams. Household paracetamol stocks were described as totals per household (in grams of paracetamol), and by type of product (formulation, means of obtaining). Households were classified into household totals of no stock, under 10 g, 10 g to under 30 g, and 30 g and more, for description of proportions of different types of products.

Statistical analysis of proportions of various stock: Using 100 replicates, multiple imputation by chain random forests and predictive mean matching was used to deal with 36 missing values for stocks of expired paracetamol, using the R package missRanger [29–31]. Population estimates of proportions were obtained by adjusting for sampling stratification on level of deprivation (1–3, 4–7 or 8–10) and city (Auckland or Dunedin), but were not adjusted for meshblock clustering, due to the low numbers of observations. The adjustment was achieved by calculating a weighted average of the proportions in each stratum, with weights equal to the product of a factor for level of deprivation (30%, 40% and 30% for 1–3, 4–7 and 8–10, respectively) and a factor for city (the number of eligible meshblocks in Auckland or Dunedin over the total number of eligible meshblocks, given a particular level of deprivation). The 95% confidence intervals (95% CI) were calculated using Wilson score interval with continuity correction on the adjusted estimates of proportions [32, 33]. For types of paracetamol stocks with missing values (‘expired’ and ‘any product’), proportions and confidence intervals were obtained by combining estimates and their variances for each of the 100 replicates using Rubin’s rules [34], and calculating the Wilson score intervals with continuity correction for the combined estimates.

## Results

A total of 201 households were recruited. The response rate in Auckland was 32.6% (100 households out of 307 consented), and 60.5% in Dunedin (101/167 consented), for a total response rate of 42.4% (201/474 consented). The participating 201 households are described in Table 1. There was a median of three usual residents per household (IQR 2–4), of which one was female (IQR 1–2) and one was male (IQR 1–2).

**Table 1:** Describing the participating households.

	**Households; n(% of 201)**
**Location**	
Auckland	100 (49.8%)
Dunedin	101 (50.2%)
**Area deprivation (NZDep2013)**	
1–3	60 (29.9%)
4–7	80 (39.8%)
8–10	61 (30.3%)
	**Households with at least one matching person; n(% of 201)**
**Sex**	
Female	185 (92.0%)
Male	173 (86.1%)
Unknown	1 (0.5%)
**Age group**	
0 to 5	39 (19.4%)
6 to 12	41 (20.4%)
13–19	34 (16.9%)
20–64	158 (78.6%)
65+	59 (29.4%)
Unknown age	8 (4.0%)
**Ethnicity**	
Māorii	17 (8.5%)
Pasifika	19 (9.5%)
Asian	29 (14.4%)
NZ European/Pākeha	141 (70.1%)
Other	25 (12.4%)
Multiple ethnicities	10 (5.0%)
Unknown	1 (0.5%)

The 201 households had a combined total of 8,757.9 g of paracetamol, of which 8,247.8 g (94.2%) was solid formulation (including powder sachets and effervescent tablets), and 510.1 g (5.8%) was liquid (Table 2). The majority of this stock was prescribed products (78.2%).

**Table 2:** Total combined load of paracetamol products in the 201 households.

Type of paracetamol product	Total load (% of total)
Solid formulation	8,247.8 g (94.2%)
Liquid formulation	510.1 g (5.8%)
Combination product	1,330.7 g (15.2%)
Expired	575.5 g (6.6%)
Prescribed product	6,845.4 g (78.2%)
Pharmacy OTC product	1,006.4 g (11.5%)
Other retailer-sourced	641.8 g (7.3%)
Other source	5.0 g (0.1%)
Unknown source	259.3 g (3.0%)
Total	8,757.9 g (100.0%)

A total of 27 study households (13.4% of 201) had no paracetamol, while 174 (86.6%) had at least one paracetamol-containing product. Based on the study sample, 19% of New Zealand households (95% CI [0.14, 0.26]) had between 10 g and under 30 g of paracetamol, while 53% (95% CI [0.45, 0.60]) had 30 g or more (Table 3).

**Table 3:** Proportions of New Zealand households with threshold stocks of paracetamol products.

	Amount of paracetamol per household; proportion* of NZ households [95% CI]				
**Type of paracetamol**	**No stock**	**Any amount >0g**	**Over 0g to <10g**	**10g to <30g**	**30g and over**
Any product	0.15 [0.10, 0.20]	0.85 [0.80, 0.90]	0.13 [0.09, 0.19]	0.19 [0.14, 0.26]	0.53 [0.45, 0.60]
Expired	0.76 [0.69, 0.82]	0.24 [0.18, 0.31]	0.14 [0.09, 0.20]	0.03 [0.01, 0.07]	0.07 [0.04, 0.12]
Combination product	0.68 [0.61, 0.74]	0.32 [0.26, 0.39]	0.17 [0.12, 0.23]	0.09 [0.06, 0.15]	0.06 [0.03, 0.10]
Prescribed	0.35 [0.29, 0.43]	0.65 [0.57, 0.71]	0.12 [0.08, 0.17]	0.17 [0.12, 0.23]	0.36 [0.29, 0.43]
Pharmacy OTC	0.79 [0.73, 0.84]	0.21 [0.16, 0.27]	0.09 [0.06, 0.14]	0.06 [0.03, 0.10]	0.06 [0.03, 0.11]
Other retailer	0.79 [0.73, 0.85]	0.21 [0.15, 0.27]	0.11 [0.07, 0.16]	0.09 [0.06, 0.14]	0.01 [0.00, 0.04]
Other source	N/A**	N/A**	N/A**	N/A**	N/A**
Unknown source	0.83 [0.77, 0.88]	0.17 [0.12, 0.23]	0.11 [0.07, 0.17]	0.04 [0.02, 0.08]	0.02 [0.00, 0.05]

NZ = New Zealand. CI = confidence interval.

*Corrected for stratified sampling (city and level of deprivation).

**One data point only; unable to calculate.

Individual households had a median of two paracetamol-containing products (IQR 1–3; range 0–15). They had a median of 24.0 g of paracetamol present per household, including 22.1 g solid products, and 6.0 g prescribed products (Table 4).

**Table 4:** Paracetamol stocks per individual household.

Type of paracetamol product	Median amount (IQR)	Range
Solid formulation	22.1 g (5.0–49.0 g)	0–593.0 g
Liquid formulation	0 g (0–0 g)	0–41.5 g
Combination product	0 g (0–4.0g)	0–214.5 g
Expired stock	0 g (0–0 g)	0–105.0 g
Prescribed product	6.0 g (0–45.0 g)	0–610.5 g
Pharmacy OTC product	0 g (0–0 g)	0–84.0 g
Other retailer-sourced	0 g (0–2.5 g)	0–82.0 g
Other source	0 g (0–0 g)	0–5.0 g
Unknown source	0 g (0–0 g)	0–41.0 g
Total	24.0 g (6.0–54.0 g)	0–610.5 g

IQR = inter-quartile range.

The 174 households which had at least one paracetamol product had a combined total of 435 products. The purchase date was known for 177 products (40.7%), and these products had been in use for a median of 120 days (IQR 37–378 days; range 0–2,285 days). A total of 342 solid products (92.4% of all 370) were of 500 mg strength, while only four (1.1%) were modified release products. A median of 24 dosing units of a solid formulation product had been originally purchased (IQR 20–100 dosing units; range 8–720), and there was a median of 18 dosing units left at the time of data collection (IQR 8–50; range 1–720). A total of 52 liquid products (80.0% of all 65) were of 250 mg/5 ml strength, while 10 (15.4%) were 120 mg/5 ml, and three (4.6%) were of unknown strength. A median of 200 ml of a liquid product had been purchased originally (IQR 200–300 ml; range 60–1,000 ml), and there was a median of 155 ml (IQR 95–200 ml; range 10–750 ml) left at the time of data collection.

## Discussion

Two significant aims of the study were achieved. Firstly, the quantities of paracetamol stock in a sample of urban New Zealand households were determined. Only 27 of the 201 households surveyed had no paracetamol products, highlighting the high prevalence of this medication being present in homes. Based on the study sample, proportions of stock in all New Zealand households were estimated. A fifth of all New Zealand households had at least 10 g but less than 30 g of paracetamol, while just over half had 30 g or more. There was much variation between individual households in stocked amounts. Secondly, the sources of this paracetamol stock were determined. The bulk of the paracetamol total mass in the combined study sample and in the individual households which were sampled was prescribed paracetamol. Based on the sample, a third of all New Zealand households had 30 g or more of prescribed paracetamol present. This is perhaps not surprising, as the co-payment for residents of New Zealand is NZ$5.00 (~US$3.50) for up to 720 units of 500 mg tablets in one prescription dispensing, corresponding to 90 days’ treatment and 360 g of paracetamol in total. A person may choose to have less dispensed, but due to the low cost of a single co-payment they may be likely to have the whole prescribed amount dispensed at once, rather than choose to pay the co-payment again at a separate dispensing event. Needs assessment by prescribers is therefore very important, to determine how much should be prescribed If a patient already has stock at home, for example, they may not need another prescription, or the newly prescribed amount could be smaller [35, 36]. This study found that a median of 24 dosing units of paracetamol (prescribed and other type) were obtained at one time, which appears to indicate that very large single purchases were not common at least in this unselected sample of urban households. Unnecessary accumulation of unused medicines at home should be avoided when possible. While from our cross-sectional study sample we are unable to investigate trends in paracetamol use in New Zealand over time, there have been increases in general in analgesic use in Germany and Norway, for example, especially in OTC product use [37, 38], and in paracetamol use in the Nordic countries and the United States [39, 40]. Increased availability through allowing sales outside of pharmacies did lead to increased incidence of paracetamol intoxications in Sweden [41], but OTC pack size reductions in the United Kingdom have not reduced deaths from paracetamol exposure [42]. Due to the way prescription medicines are subsidized in New Zealand there may be more prescription paracetamol stock present in households at any given time, as found in the present study.

In the United Kingdom, changes in policy and practice to reduce paracetamol availability have concentrated on OTC access [43]. In New Zealand pack size available OTC is limited but there is no restriction on the number of packs that can be purchased at once. National sales figures of OTC paracetamol are not collected in New Zealand, and cannot therefore be used to assess the extent of such purchases and level of population exposure to OTC products in the context of harm from paracetamol used in ISP or in unintentional overdoses. Our study suggests that in New Zealand, the current policy priority should be to focus on limiting prescription paracetamol, so that people have what they need, but not so much that large stocks are built up. Monitoring OTC access through a national sales dataset could inform policy, however, and should be considered.

Our study found that 24% of households had expired paracetamol products. This is a lower rate than the two-thirds of households found in a recent Belgian study [44], and higher than the 2.4% found in a Brazilian study [45]. Initiatives such as ‘Disposal of Unwanted Medication Properly (DUMP)’ previously done in New Zealand [46] or ‘Return Unwanted Medicines (RUM)’ in Australia [35] where people had an easy, simple means of returning unnecessary medicines to the pharmacy could assist in reducing such expired stock being accumulated in households. This would assist in reducing inappropriate access such as use in intentional self-poisoning, or accidental pediatric exposures.

### Limitations

This study provides a cross-sectional view of how much stock was present at one point in time. Those who were not at home during data collection, who chose to not take part, or whose house appeared unsafe to approach were excluded from the study. People may have ‘censored’ stock they had at home, by accident or on purpose. Any non-participating household member may have had private stock which would not have been recorded. Further, since this was an urban sample, it may not describe the paracetamol stocks held at rural New Zealand households; needs among rural households for storing medicines may differ due to long distances to pharmacies or other retailers. Finally, our study only describes the situation in New Zealand–stocks in other countries may differ significantly, based on local prescribing conventions, regulations on sales, and availability of different paracetamol products.

## Conclusions

A large majority of New Zealand households have paracetamol stocks readily accessible, and these were mainly solid formulation paracetamol obtained with a prescription. Ensuring people have sufficient access to paracetamol for pain management needs to be balanced with preventing unnecessary accumulation of unused stock in households to minimize inappropriate use such as for intentional self-poisoning.
